# Cross-Sectional Melt Pool Geometry of Laser Scanned Tracks and Pads on Nickel Alloy 718 for the 2022 Additive Manufacturing Benchmark Challenges

**DOI:** 10.1007/s40192-024-00355-5

**Published:** 2024

**Authors:** Jordan S. Weaver, David Deisenroth, Sergey Mekhontsev, Brandon M. Lane, Lyle E. Levine, Ho Yeung

**Affiliations:** 1 Engineering Laboratory, National Institute of Standards and Technology, 100 Bureau Dr, Gaithersburg, MD 20899, USA; 2 Materials Measurement Laboratory, National Institute of Standards and Technology, 100 Bureau Dr, Gaithersburg, MD 20899, USA

**Keywords:** Laser powder bed fusion, Melt pool size, Optical microscopy, Model validation

## Abstract

The Additive Manufacturing Benchmark Series (AM Bench) is a NIST-led organization that provides a continuing series of additive manufacturing benchmark measurements, challenge problems, and conferences with the primary goal of enabling modelers to test their simulations against rigorous, highly controlled additive manufacturing benchmark measurement data. To this end, single-track (1D) and pad (2D) scans on bare plate nickel alloy 718 were completed with thermography, cross-sectional grain orientation and local chemical composition maps, and cross-sectional melt pool size measurements. The laser power, scan speed, and laser spot size were varied for single tracks, and the scan direction was varied for pads. This article focuses on the cross-sectional melt pool size measurements and presents the predictions from challenge problems. Single-track depth correlated with volumetric energy density while width did not (within the studied parameters). The melt pool size for pad scans was greater than single tracks due to heat buildup. Pad scan melt pool depth was reduced when the laser scan direction and gas flow direction were parallel. The melt pool size in pad scans showed little to no trend against position within the pads. Uncertainty budgets for cross-sectional melt pool size from optical micrographs are provided for the purpose of model validation.

## Introduction

The Additive Manufacturing Benchmark Series (AM Bench) is a NIST-led organization that provides a continuing series of additive manufacturing (AM) benchmark measurements, challenge problems, and conferences with the primary goal of enabling modelers to test their simulations against rigorous, highly controlled additive manufacturing benchmark measurement data [1]. Additive manufacturing is a layer-by-layer process where material is added in a layer wise fashion to achieve the part geometry. Each layer is also typically a track-by-track process where material is deposited in individual tracks to fill in a single layer. The basic building block for laser based additive manufacturing of metals is a single-track laser scan (i.e., a line scan), which can be used to determine the melt pool size under various process parameters. The term “melt pool” in this context is material that was once liquid and has resolidified. Single-layer and single-track experiments are useful measurements for model validation (e.g., AM Bench 2018 [2] and Air Force Research Lab (AFRL) challenge series [3]), process development (e.g., [4]), and process control and qualification (e.g., [5]). The present single-track and single-layer experiments are from the 2022 AM Bench measurements and challenges [6] and focused on melt pool size measurements from cross-sectional optical microscopy.

The 2022 AM Bench measurements built upon the previous 2018 measurements by expanding from single-track laser scans to also include single-layer scans (i.e., 2D geometries or pad scans). The single-track and pad scans are produced on bare plates without powder feedstock. The bare plate experiments are simpler for rigorous measurements and modeling. Changes in the laser power, scan speed, and laser spot size were included in single-track experiments as these are the most influential laser processing parameters. Single tracks lack aspects of remelting and heat buildup that occur in a layer while pad scans include these aspects. The pad geometry and scan strategy were chosen to replicate an individual layer in the related 3D build measurements and challenges. In addition to cross-sectional melt pool size measurements for the single-track and pad scans, infrared thermography and microstructure measurements via electron backscatter diffraction (EBSD) and energy-dispersive spectroscopy (EDS) were also taken on the same single-track and pad scan samples. As previously mentioned, the 3D build contains layers related to the pad scans. Infrared thermography and microstructure measurements are also available for the 3D build. The only difference in laser parameters between the pad scan and 3D build is that the laser spot size (D4*σ*) was 77 μm for the 3D builds and 67 μm for the pad scans. The related datasets and journal publications are listed in Table 1. This paper focuses on results from optical microscopy.

## Methods

### Material and Laser Processing

Nickel alloy 718 plate with a thickness of 3.17 mm (1/8 in.) was cut into 25.4 mm × 25.4 mm (1 × 1 in.) pieces. The plate chemistry provided by the manufacturer is listed in Appendix 1. The plates were ground with 320 grit SiC paper. The typical resulting surface roughness as determined from stylus-type surface profiler measurements was Ra = 0.15 μm (5.8 μin). Laser tracks and pads were made using the NIST Additive Manufacturing Metrology Testbed (AMMT) [16]. The base process conditions for single-track scans are listed in Table 2. In addition to the baseline laser parameters, three cases that changed the laser spot size, speed, and power were included. These cases and the corresponding laser parameters are listed in Table 3.

**Fig. 1:**
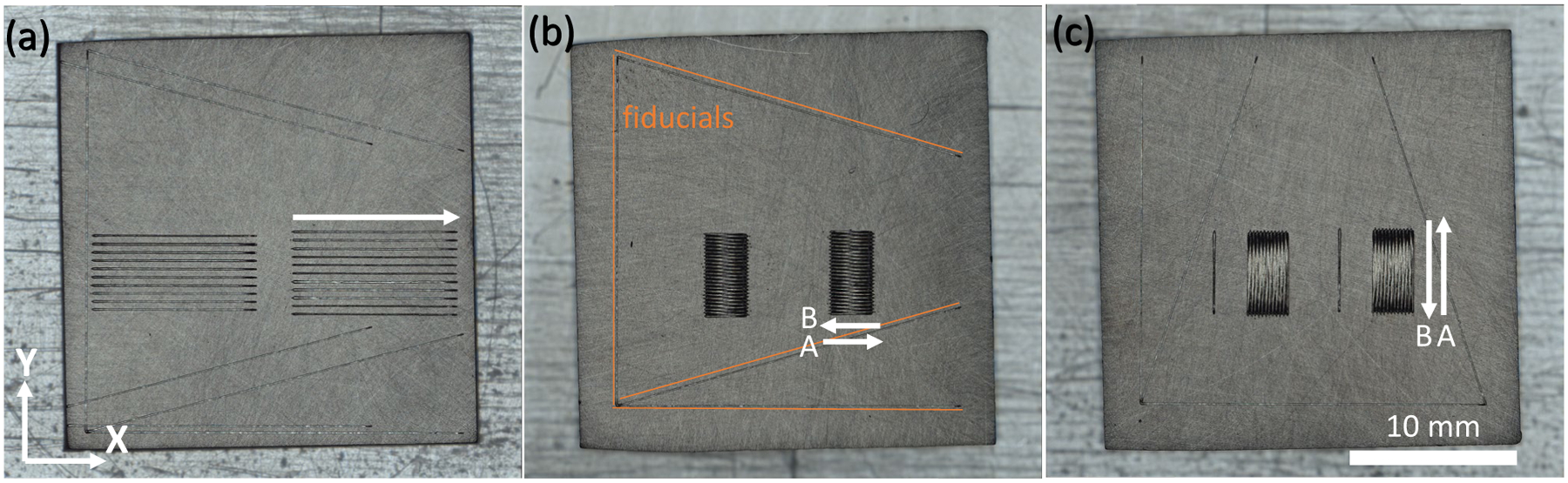
Top view images of **a** single track, **b** X-pad, and **c** Y-pad plates. Arrows indicate the laser scan direction. These three plates are referred to as AMB2022–718-SHI-BP1, AMB2022–718-SHI-BP2, and AMB2022–718-SHI-BP3, respectively, for the AM Bench challenge problems [18]

The pad scans were produced with the baseline laser parameters using a hatch spacing (spacing between laser tracks) of 110 μm. The 2D pad geometry was fixed at 2.5 mm in the *x*-direction and 5 mm in the *y*-direction. The X-pad and Y-pad experiments were designed to replicate the odd and even layers in the 3D build bridge structure (see Table 1). The sample reference frame is based on the laser powder bed fusion (L-PBF) machine reference frame: Recoating axis is along the *x*-direction, the y-direction is from the front of the machine to the back, and the building direction is the *z*-direction [17]. The laser scan direction was in the ± *x*-direction for the X-pad and ± *y*-direction for the Y-pad. The first track of the X-pad was in the positive *x*-direction (A), and the second track was in the negative x-direction (B). This AB pattern is repeated to fill in the pad geometry. The time between each track (i.e., the laser turnaround time) was ≅ 40.2 ms on the A to B side and ≅ 5.3 ms on the B to A side. The longer turnaround for one side of the X-pad is caused by the fact that the laser also fills in two additional legs of the 3D build geometry. These were not printed during the X-pad experiments; however, the exact same laser path and timing was used to replicate the 3D build. The annotated laser path is provided in Appendix 2. The first track of the Y-pad is in the positive *y*-direction (A), and the second track is in the negative *y*-direction (B). Again, the AB pattern repeats with a laser turnaround of ≅ 5.3 ms on both the A to B and B to A sides of the pad. This results in 47 tracks for the X-pad and 23 tracks for the Y-pad. Two of each pad were generated per plate. Top view images of the single tracks and pads are shown in Fig. 1. Additional laser tracks were added as fiducials.

### Cross-Sectional Measurements

**Fig. 2:**
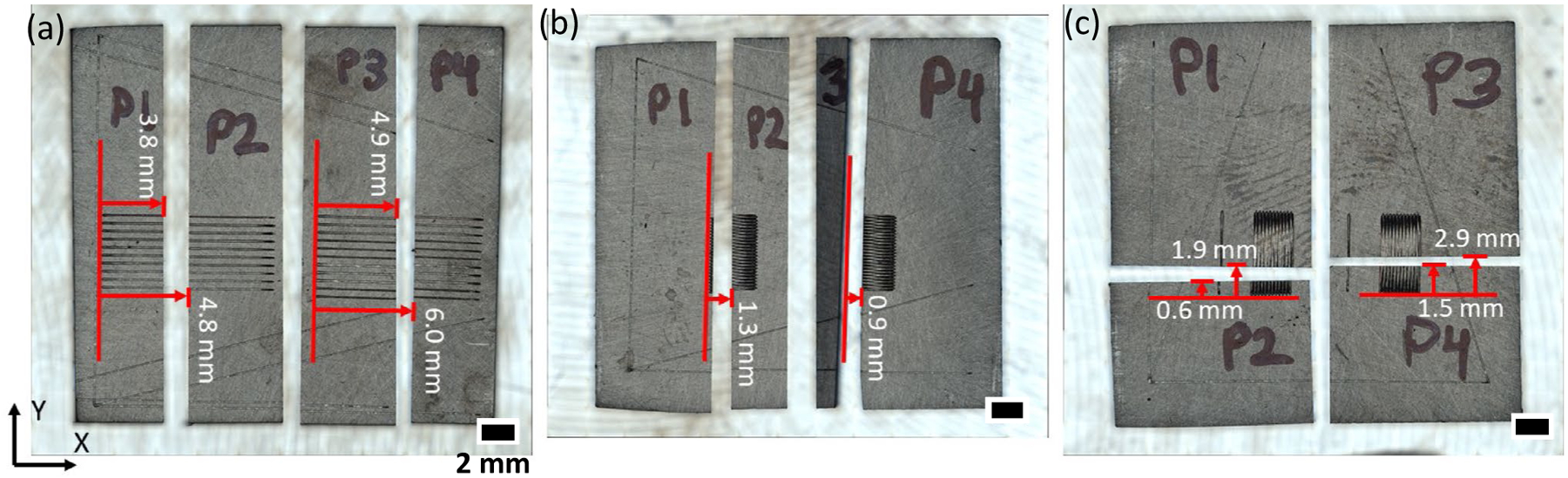
Top view images of **a** single track, **b** X-pad, and **c** Y-pad plates after cross-sectioning. The positions of cross sections listed on each part were determined after metallographic sample preparation using fiducial markings. Red arrows are shown for schematic purposes only and do not indicate measurements from these images. Each piece of the plate has a suffix of P1, P2, P3, or P4 for the AM Bench challenge problems naming convention (e.g., AMB2022–718-SHI-BP1-P1)

Plates were cross-sectioned perpendicular (within 2°) to the laser scan direction. Single tracks were cross-sectioned in approximately the middle to determine the steady state melt pool size, and pad scans were cross-sectioned in multiple locations to assess the positional dependence of the melt pool geometry. Top view images of the cross sections are shown in Fig. 2, which also includes the cross-sectional positions. Fiducial lines were included to establish the cross-sectional positions. The distance between fiducial lines on cross sections was measured on metallographically prepared and etched samples, and the position of the plane was calculated assuming the cross section is aligned along one of the Cartesian axes. The standard uncertainty (*k* = 1, type B) of the cross-sectional position is estimated to be ± 0.2 mm. The samples were mounted and metallographically prepared followed by etching with aqua regia to reveal the melt pool boundaries. Optical microscopy was used to evaluate the melt pools. Dark-field images were taken at 500 × magnification with a pixel scaling of 0.069 μm per pixel. Multiple images were stitched together for melt pools that extended beyond a single field of view. Geometry measurements were taken in ImageJ.^1^

**Fig. 3:**
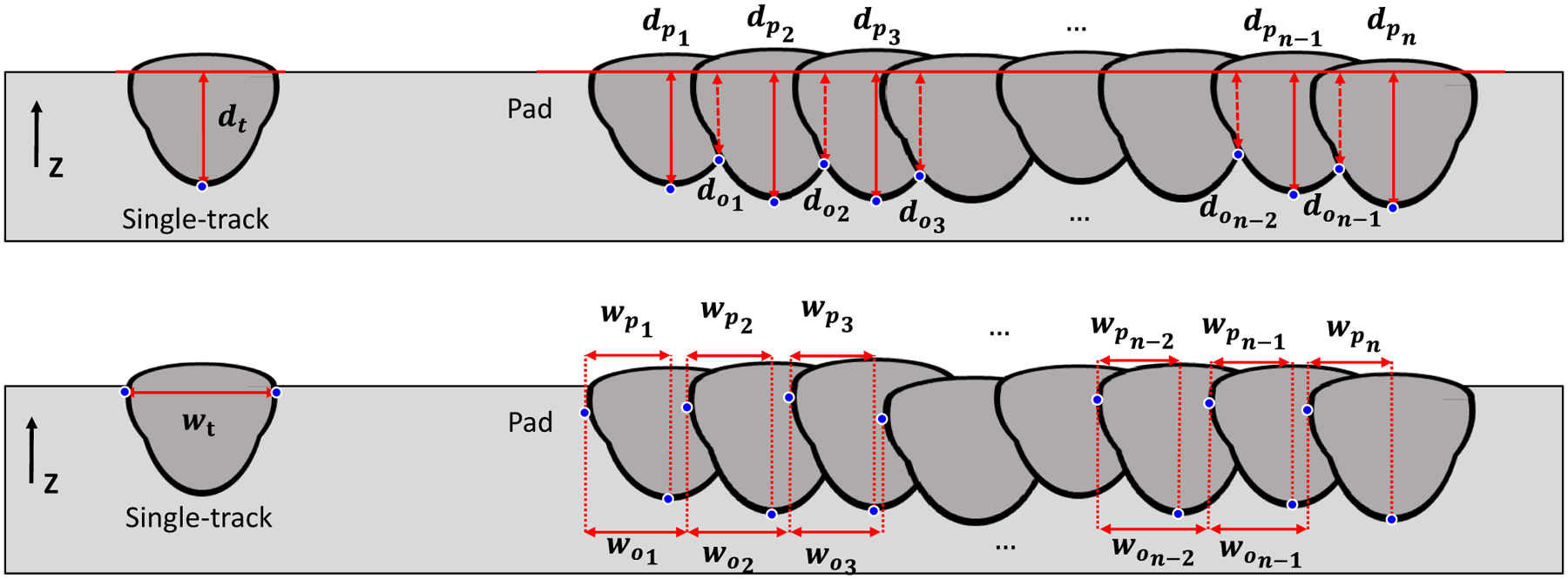
Melt pool depth and width measurement definitions shown schematically. Single-track depth, *d*_t_, and width, *w*_t_ . Pad scan depth, *d*_p_, and width, *w*_p_, and the overlap depth, *d*_o_, and *w*_*o*_, width. The pad depths and widths count from 1 to the total number of tracks, *n*. The pad overlap depths and widths have a total of *n* − 1 measurements

Melt pool depth and width measurements are shown schematically in Fig. 3. These definitions are not universal nor are these the only important features in the melt pool morphology. These were chosen for simplicity to easily compare experiments and models while capturing the general behavior. Because there is no powder in these experiments, the melt pool depths are defined from the top surface of the plate, ignoring any humping of material above the starting surface. Depth measurements are perpendicular to the top surface, and width measurements are parallel to the top surface. The depths, *d*_t_ and *d*_p,_ are are always the largest vertical distance, not necessarily in the center of the melt pool. The subscripts *t* and *p* refer to the single-track and pad measurements, respectively. The pad overlap depth, *d*_o_, is the distance from the top surface to where melt pools intersect. The widths are always the widest horizontal distance, not necessarily at the top surface. The pad track width, *w*_p_,is measured from the depth line, *d*_p_, to the widest point of the melt pool. The pad overlap width, *w*_*o*,_ is the distance between the widest point of a melt pool and the subsequent melt pool. (e.g., $w_{o_{1}}$ is measured between track 1 and track 2). These points need not be in the same Z-plane. Due to the remelting in pad scans, pad track width, *w*_p_, is approximately half the width of the melt pool and should be multiplied by 2 to compare with single-track widths.

### Challenge Predictions

The measurements were part of the AM Bench 2022 challenge series where modelers were provided key details of the experiments and asked to predict the average melt pool depths and widths [18]. A total of eight submissions were received for predicting single tracks, and four submissions were made for the pad scans. The methodology for the predictions was not part of the submissions. The predictions have been anonymized. As such, the comparison to models in this manuscript should be seen as bird’s-eye view rather than an evaluation of specific models and assumptions.

## Results

### Single Tracks

**Fig. 4:**
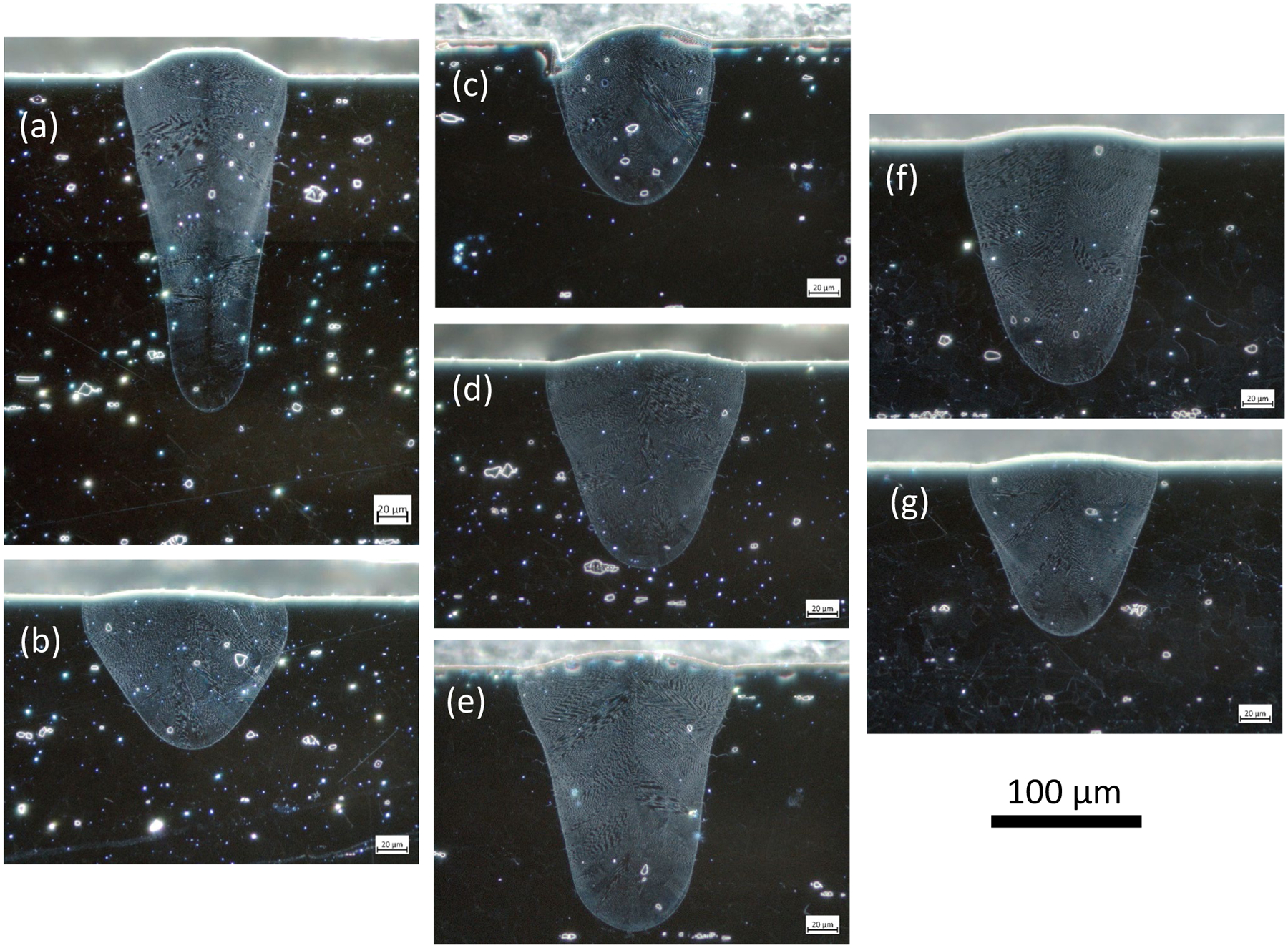
Dark-field micrographs of melt pool cross sections for the six cases: **a** case 1.1, **b** case 1.2, **c** case 2.1, **d** case 0, **e** case 2.2, **f** case 3.1, and **g** case 3.2

**Fig. 5:**
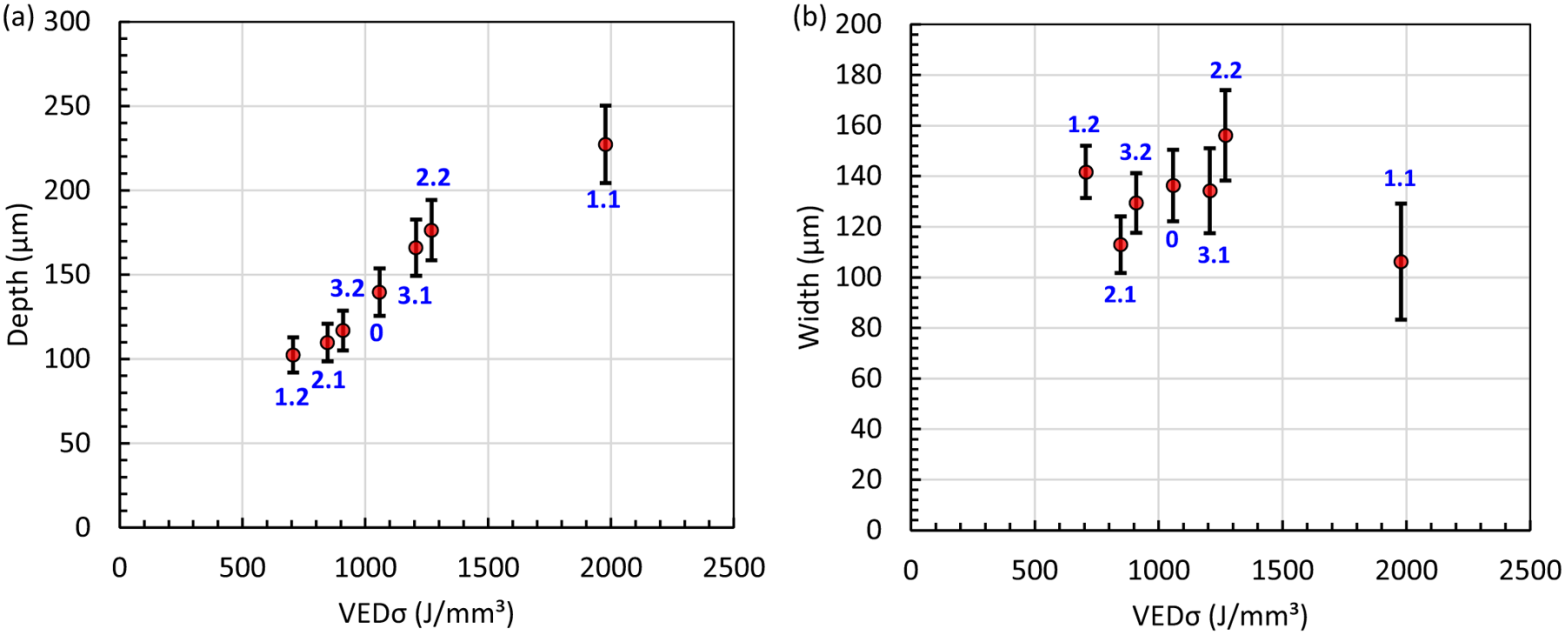
Single-track **a** depth and **b** width measurements. Data points are the mean ± U (*k* = 2). The individual case numbers are labeled in blue

Representative micrographs for the seven cases with different laser power, scan speed, and laser spot size are shown in Fig. 4. The width and depth measurement results are provided in Table 4 and plotted in Fig. 5. The laser power, *P*, scan speed, *v*, and 1*σ* of the laser spot diameter*(D*4*σ/4* = *σ*) can be combined into a single term known as the volumetric energy density (VED*σ = P/v/σ*^2^) The *σ* in VED*σ* denotes that the formula uses the laser spot size and not the more common formula based on 3D building parameters (i.e., hatch spacing and layer thickness). There is a general trend of increasing melt pool depth with increasing VED*σ*, whereas the melt pool width shows no apparent trend with VED*σ* as shown in Fig. 5. Here, we note that the lowest and highest VED*σ* are produced by the largest (case 1.2) and smallest (case 1.1) laser spot size, respectively. Increasing the laser spot size broadens the power distribution, lowering the VED*σ* while creating a more shallow and broader melt pool. The opposite is true of decreasing the spot size: a narrower power distribution increases the VED*σ* while producing a deeper and narrower melt pool. Melting modes are commonly categorized as conduction, transition, and keyholing where the transition between conduction and keyholing starts when the ratio of the depth to half width (i.e., aspect ratio) becomes greater than 1. Here we note that all the cases have an aspect ratio greater than 1.

### Pad Trends

**Fig. 6:**
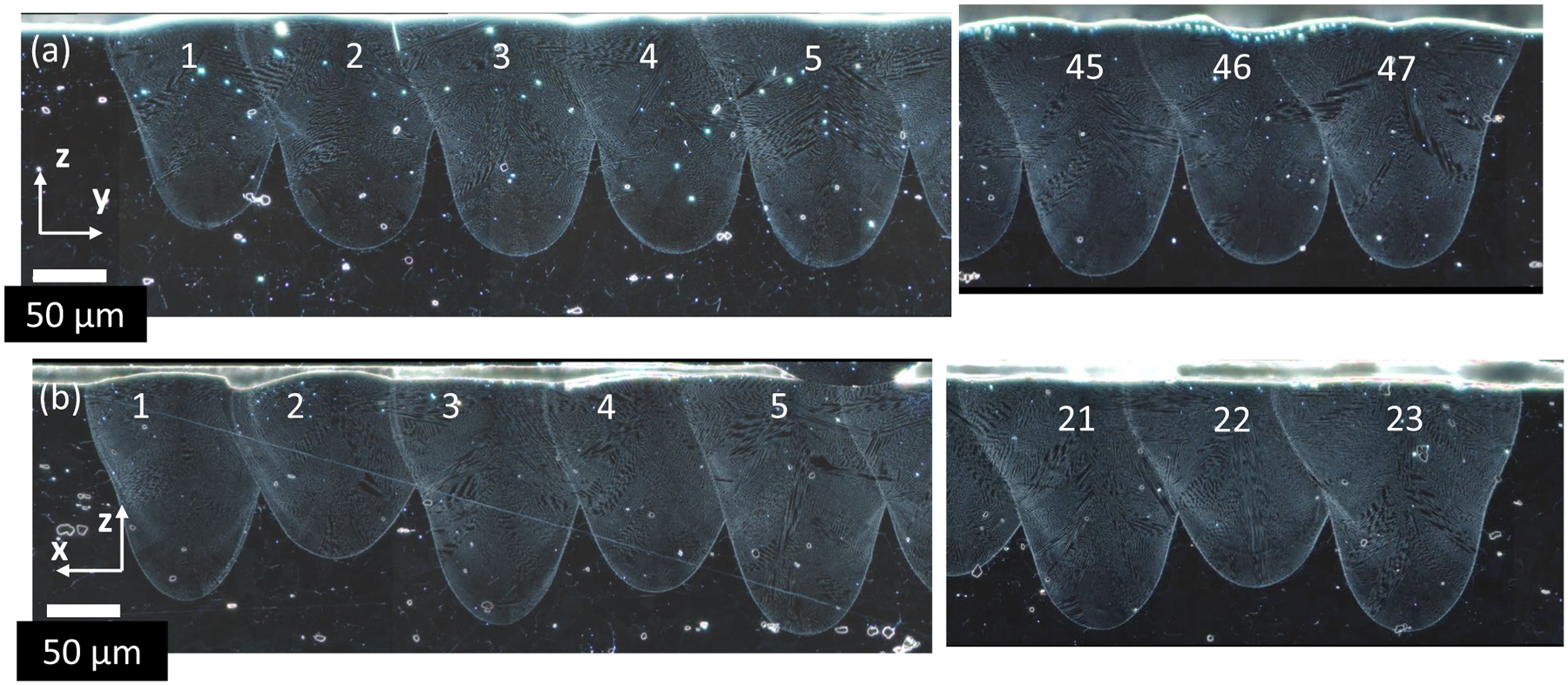
Dark-field micrographs of melt pool cross sections for **a** X-pad and **b** Y-pad. The micrographs show the first five and last three tracks of each pad. The X-pad and Y-pad are AMB2022–718-SHI-BP2-P2 and AMB2022–718-SHI-BP3-P3, respectively

**Fig. 7:**
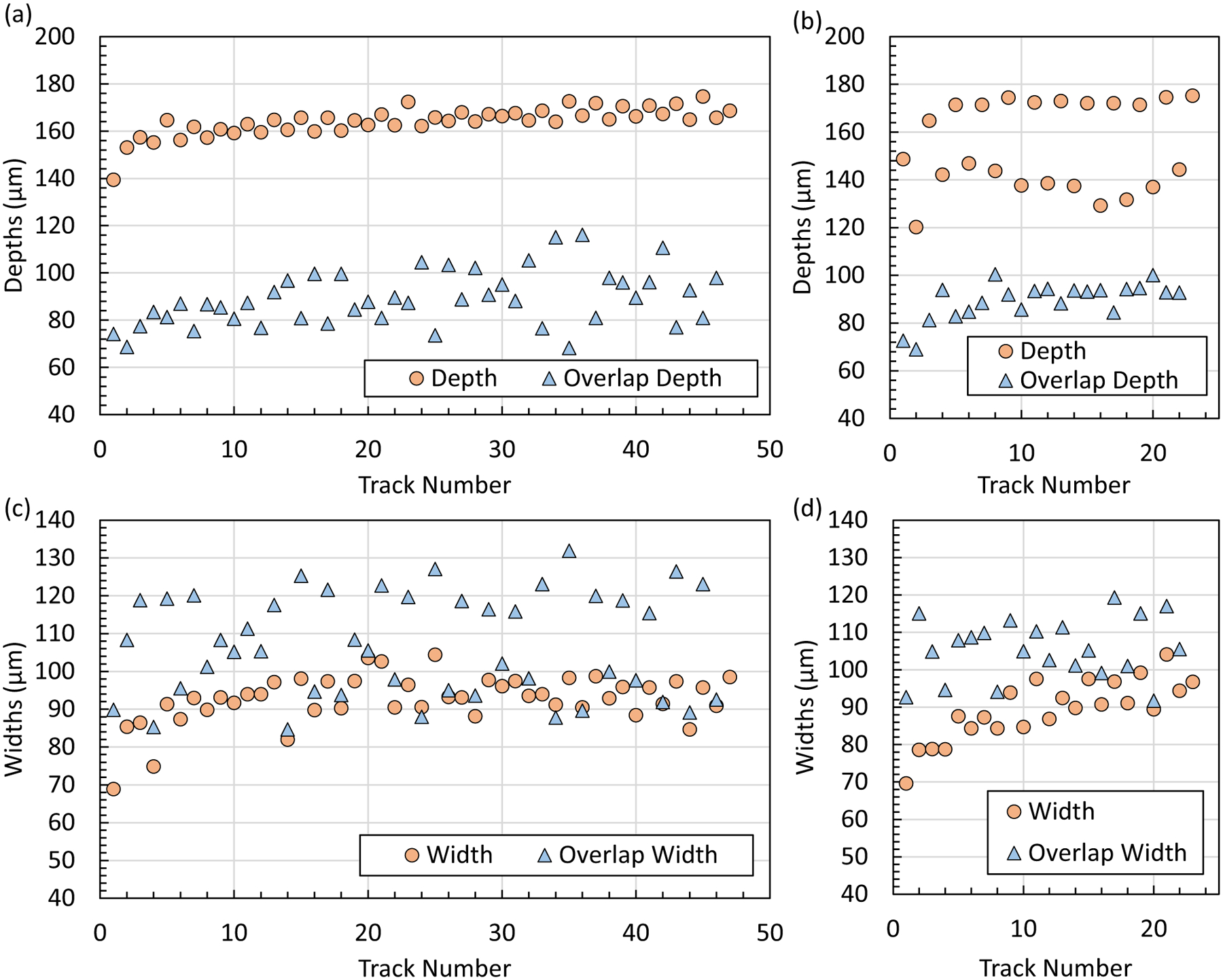
Pad scan melt pool measurements for X-pad (AMB2022–718-SHI-BP2-P2) and Y-pad (AMB2022–718-SHI-BP3-P3). **a** X-pad depths, **b** Y-pad depths, **c** X-pad widths, **d** Y-pad widths. Refer to Fig. 3 for measurement definitions. Note the width definition for a pad is analogous to a half width for single track. A type B uncertainty of ± 3 μm (*p* = 95%) is estimated to account for user selection and microscope resolution for the purpose of comparing different track numbers on a single cross section. This is approximately the size of the data points

The pad scan melt pool results contain multiple characteristics: trends with track number, X vs. Y scan direction, and trends with cross-sectional position within each pad. Figure 6 shows representative micrographs of the X-pad and Y-pad for the first five tracks and last three tracks for cross sections taken close to the center of each pad. Figure 7 shows the melt pool depths and widths versus track number for the same cross sections in Fig. 6. As a reminder, the pad geometry is 2.5 mm in the x-direction and 5.0 mm in the y-direction, which results in a different number of tracks for X and Y pads. There are several observations from the micrographs in Fig. 6 and measurements in Fig. 7. First, the Y-pad differs from the X-pad in that the Y-pad depth shows a decrease in depth every other track while the depth does not change significantly between tracks in the X-pad (Figs. 6b and 7b). In other words, even track numbers (laser scan direction is − Y) have a shallower depth than odd track numbers (laser scan direction is + Y). This correlates with the laser scanning parallel and anti-parallel to the − Y gas flow direction, respectively. The parallel configuration of scan direction (− Y) and gas flow direction (− Y) can lead to an increase in interaction between the by-product plume (especially condensed nanoparticles) and laser causing attenuation, scattering, and/or lensing [20, 21]. Other than this, the X-pad and Y-pad (odd track numbers) have comparable depths and widths.

Second, there is a clear increase in melt pool depth over the first three to five tracks for both the X-pad and Y-pad (neglecting the odd/even pattern), as shown in Fig. 7a and b. After the fifth track, the X-pad depth gradually increases with track number, whereas the Y-pad depth shows no trend with increasing track number beyond the start (neglecting the odd/even pattern). The width also appears to be increasing with track number over the first 10 tracks for both pads (Fig. 7c and d). After 20 tracks, the trend in width with track number flattens out for the X-pad (the Y-pad only contains 23 tracks). The overlap width and overlap depth tend to have more variability than the depth and width. The overlap width is scattered about the hatch spacing of 110 μm, which is expected because this measurement is akin to the track spacing. There were no repeat measurements at the same cross-sectional position from which to derive a mean and an uncertainty estimate for each track number. A type B uncertainty of ± 3 μm (*p* = 95%) is estimated to account for user selection and microscope resolution for the purpose of comparing different track numbers on a single cross section. The general trends with track number mentioned above were observed for all six of the cross sections in this work.

**Fig. 8:**
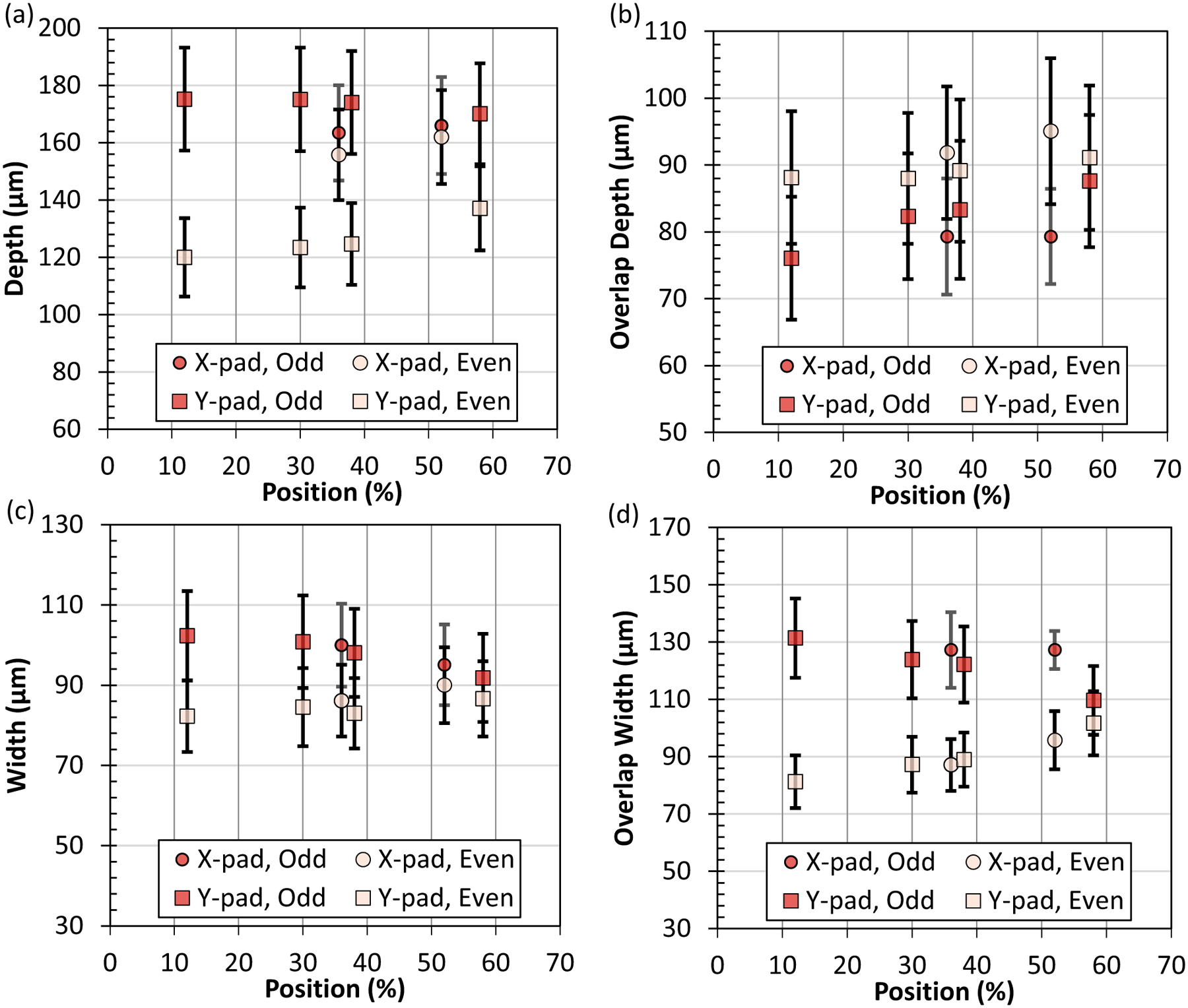
Pad scan melt pool results for **a** depth, **b** overlap depth, **c** width, **d** overlap width separated by X-pad/Y-pad and odd/even track numbers. The x-axis is the relative position normalized by the pad with for each scan direction: 2.5 mm for the X-pad and 5.0 for the Y-pad. A value of 50% is the middle of the pad. Data points are the average ± U (*k* = 2). See Appendix 4 for values of U

The last characteristic of the pad scans is the possible change in melt pool size with position along the tracks. The averages for each cross-sectional position and odd or even numbered track groups are listed in Table 5. The measurement results are also plotted in Fig. 8 versus the relative track position. The X-pad and Y-pad relative positions are normalized by the pad widths of 2.5 mm and 5.0 mm, respectively, so they can be plotted together. A relative position of 50% is the middle of the pad. A relative position near 0% approaches the starting edge of the pad. Along the starting edge, odd tracks scan away from the edge and even tracks scan toward this edge. Near this edge, there is a potential for the melt pool of even tracks to still be liquid or a very hot solid when the odd tracks start. This scenario switches at the other edge of the pad. This is one of the reasons for the grouping of odd and even track numbers in this analysis. The other reason was the previously mentioned Y-pad odd/even trend corresponding to the gas flow direction. Figure 8 reveals any trends in melt pool size with position.

The pad depth, overlap depth, width, and overlap width versus position are each presented in Fig. 8. The behavior for the Y-pad depth (smaller depth for even tracks compared to odd tracks) is present for all cross-sectional positions (Fig. 8a). The depth difference between Y-pad odd and even tracks is greatest at the starting edge at a relative position, around 10%. The X-pad depth does not show a dependence on position. The overlap depth (Fig. 8b) does not show a clear trend with position for either X-pads or Y-pads. The overlap depth even track number averages are consistently deeper than their odd counterparts but with overlapping error bars. Track width (Fig. 8c) shows odd tracks with consistently greater averages than their even counterparts but with overlapping error bars. The overlap width (Fig. 8d) shows a clear trend with position. The odd overlap width decreases as the relative position increases, and the even overlap width increases as the relative position increases until the odd and even groups overlap. Some possible explanations for these observations will be provided in the discussion.

### Prediction Trends

**Fig. 9:**
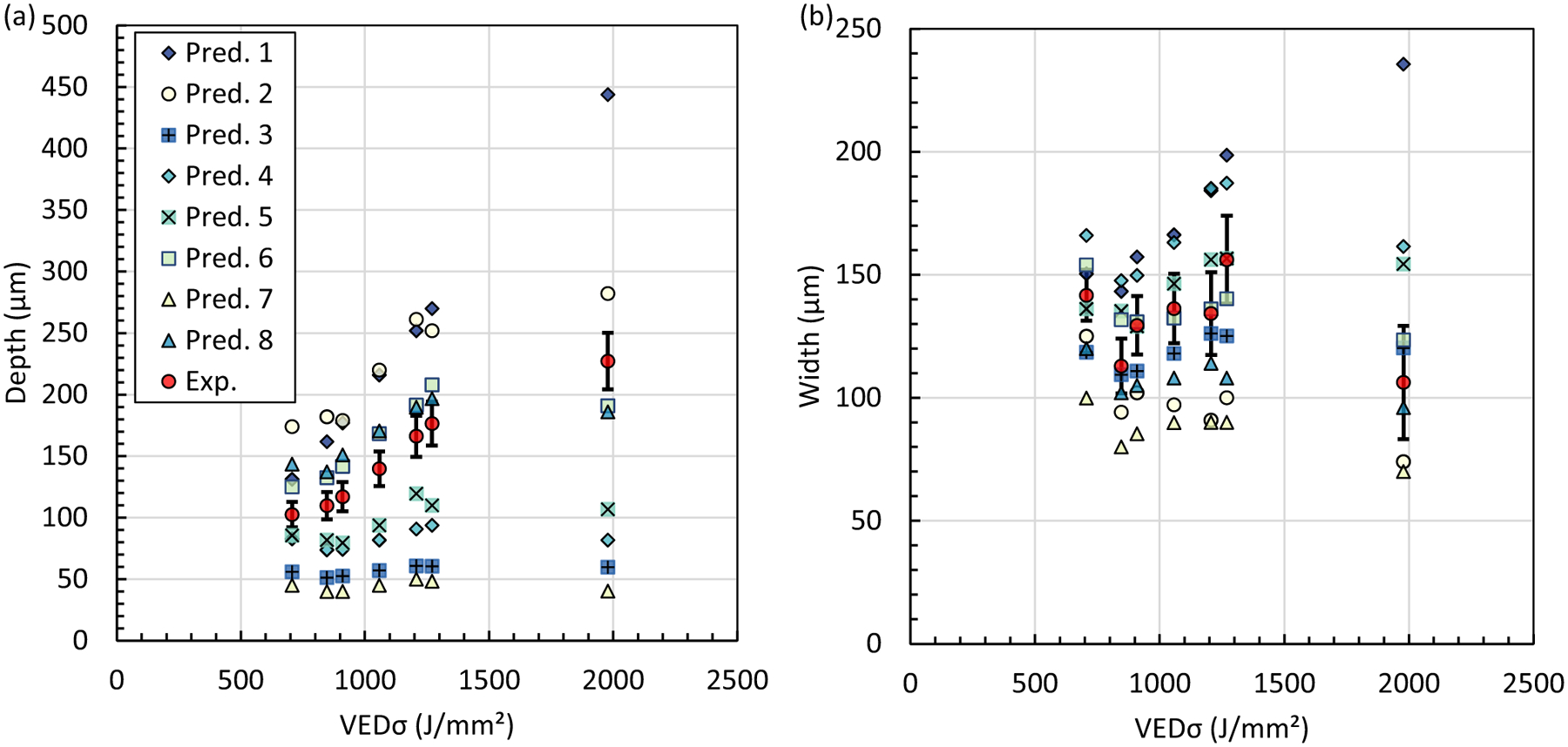
Single-track average melt pool measurements (Exp.) and predictions (Pred. #) for **a** depth and **b** width versus the volumetric energy density (VED*σ*). The error bars on the measurements are ± the combined, expanded uncertainty, U (*k* = 2)

The single-track melt pool average depth and average width measurements (Exp.) and predictions (Pred. #) are plotted in Fig. 9 as a function of VED*σ*. Depth and width predictions fall above and below the measurements. A few depth predictions follow the trend with VED*σ*; however, very few case predictions fall inside the expanded (*k* = 2) measurement uncertainty. On the other hand, several width predictions fall inside the measurement uncertainty. The range for the eight predictions for depth is the greatest for the case with the highest VED*σ*. The melt pool size is highly dependent on how much of the laser energy is absorbed. The absorption is not constant and depends on the laser melting mode and vapor depression that forms [22, 23]. This could be one reason why there is a large spread in the predictions for depth and few predictions inside the measurement uncertainty bars.

**Fig. 10:**
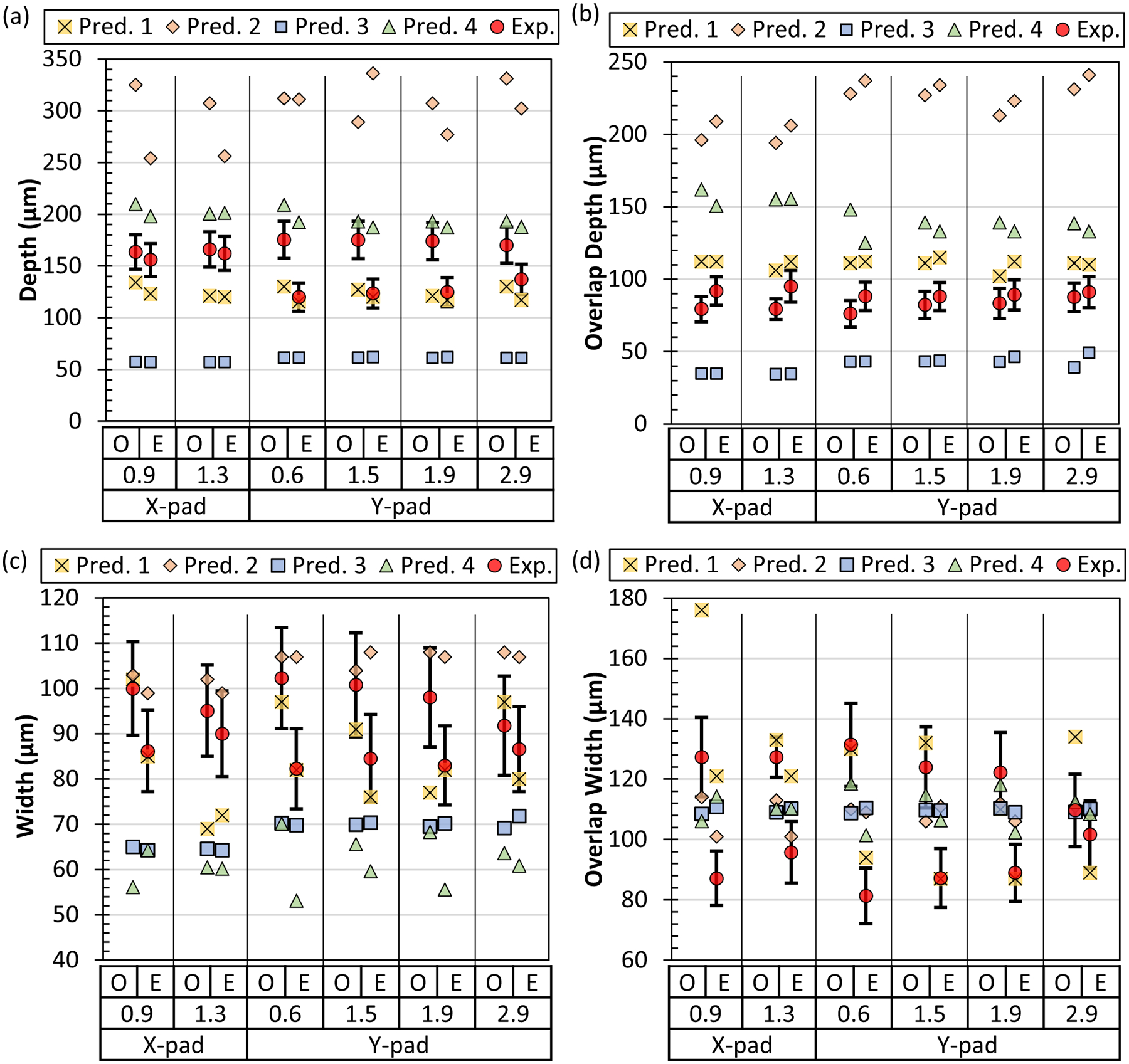
Pad scan average melt pool measurements (Exp.) and predictions (Pred. #) for each Pad cross section: **a** depth, **b** overlap depth, **c**, width, and **d** overlap width. Averages are for odd track (O) and even track (E) numbers on each cross section. The distance for each cross section is listed in millimeters. The error bars on the measurements are ± the combined, expanded uncertainty, U (*k* = 2); see Appendix 4 for uncertainty estimates. Refer to Figs. 2 and 3 for a diagram of the cross-sectional positions and melt pool measurement definitions, respectively

The measurements (Exp.) and predictions (Pred. #) for the pad melt pool measurements are shown in Fig. 10. The uncertainty budget for the average melt pool measurements for each cross section is similar to single-track measurements and detailed in Appendix 4. Modelers were asked to predict the average depth, average overlap depth, average width, and average overlap width for odd and even track numbers at each cross-sectional position. The depth predictions from the various participants bracket the measurement results, but only two groups have some results within the expanded uncertainties of some of the measurements. The difference between odd and even track numbers for the Y-pad depth (Fig. 10a) was not captured by any of the predictions. The overlap depth (Fig. 10b) predictions showed a similar spread as the depth prediction, with none falling within the measurement uncertainties. The width (Fig. 10c) and overlap width (Fig. 10d) predictions showed a tighter spread. Many width predictions were reasonably close to the measurements. The overlap width predictions show the tightest spread. This could be because the prescribed hatch spacing, 110 μm, is akin to the overlap width if the melt pools remain a constant size and shape.

## Discussion

### Single-Track Versus Pad Scans

**Fig. 11:**
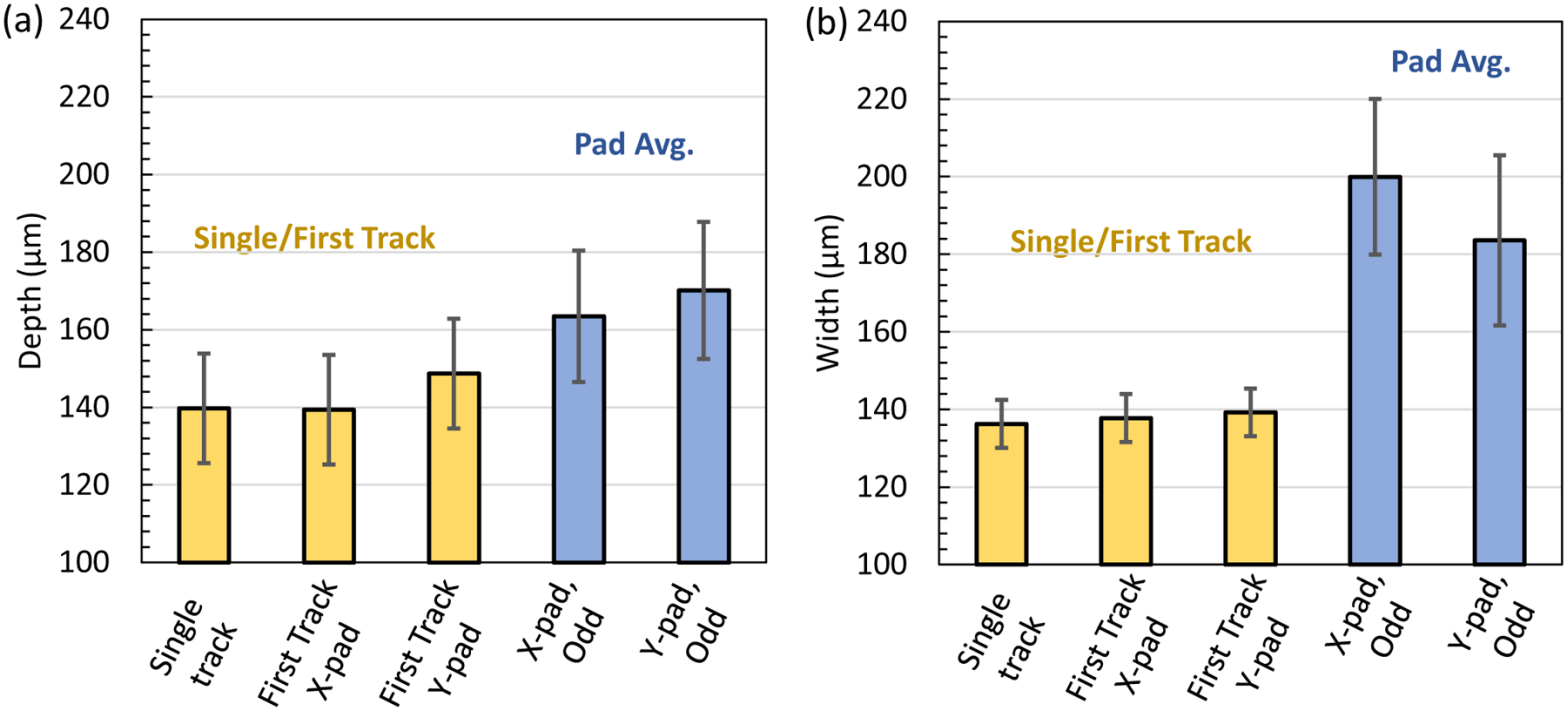
Comparison of the single track, first track in the pad scans, and the pad scan **a** depth and **b** width. The pad cross-sectional measurements come from X-pad, 1.3 mm (AMB2022–718-SHI-BP2-P2) and Y-pad, 2.9 mm (AMB2022–718-SHI-BP3-P3). The pad width measurements (half widths) were multiplied by 2 to compare with the single-track width measurement (full width). Error bars are ± U (*k* = 2)

While single tracks are highly useful for model and process development, it is clear that they do not represent the steady state or average track behavior for a layer with a 2D scan geometry. Figure 11 shows a comparison of the single track, Case 0, to the first track of the X-pad and Y-pad on cross sections near the middle of the pads. The first track of a pad is the same as a single track except that it is later partially remelted. In this case, the pad melt pool width measurement is a half width that was doubled for the comparison to a single-track width. The expanded uncertainty from repeat measurements of single tracks was applied to the individual first track pad measurements to aid in the comparison. The depths and widths are nominally the same for the single track and first pad tracks. Figure 11 also compares this group of single/first tracks to the average pad scans for odd tracks. The average depth of the pads is consistently higher; however, the difference is within the expanded uncertainty. The width of pads is significantly larger than single/first tracks. Both observations are likely due to the residual heat buildup in the plate. This is supported by thermography measurements that show an increase in the time above melt and decrease in cooling rates from the first track to the rest of the pad [9]. Hence, it is important to validate models against single tracks as well as pad scans to accurately predict melt pools in 2D layers.

### Pad Scan Trends with Position

Pad scans with an AB scan pattern tend to have variation in thermal history, which may cause a melt pool size dependence on position. The theory is that as one track finishes (B), it remains liquid or very hot solid as the next track starts (A). This causes the melt pool size and shape to change significantly on the A-tracks. Recent Air Force Research Laboratory challenge series experiments and simulations showed a difference between odd and even numbered tracks near the edge of the pad for an ± X-direction scan strategy (both directions are perpendicular to the gas flow direction) [3, 24]. Near the pad edge corresponding to the starting point for the first track, the depth and width significantly increased for odd track numbers and remained the same or slightly decreased for even track numbers. This behavior occurred when within 1 mm of the edge and was most exaggerated for a cross section within 100 μm of the edge. This has been observed by others in laser turnaround regions where “double-wide” melt pools form [25]. The AM Bench pads do not show this behavior for the limited number of cross sections. Recall the main trend for odd and even tracks was observed for the Y-pad, a reduced depth for even tracks likely caused by the increased interaction of the vapor plume with the laser caused by the parallel configuration of the scan direction and gas flow direction. This reduced depth for Y-pad even numbered tracks was consistent throughout the pad and is not the same phenomena seen in the AFRL pads near the edges of the pads. Rather, the AM Bench pads show a very modest dependence on cross-sectional position due to the laser turnaround effect. For instance, the difference between the odd and even track depth in the Y-pad is greatest near the starting edge (Fig. 8a). Additionally, the difference between the overlap width for odd and even tracks increases near the starting pad edge with the odd overlap depth greater than the even overlap depth (Fig. 8c). This indicates more remelting into the previous track for odd tracks and slightly less remelting for even tracks. This does not show up strongly in the pad width measurements but rather in the overlap width measurements because the overlap width measures the cumulative effect of over melting/under melting.

The key to explaining the different cross-sectional position trends between the AM bench pad results and the AFRL pad results is the laser turnaround time. The laser turnaround time is the time between when the power turns off at the end of one track and turns back on for the next track. The AFRL pad turnaround time is estimated to be approximately 0.5 ms for the commercial machine used in the experiments. The turnaround time for the experiments on the AMMT were an order of magnitude larger at values of ≅ 5.3 ms and ≅ 40.2 ms depending on the specific pad type and edge. The much larger laser turnaround time means the track going into the edge likely solidifies and cools somewhat before the next track starts and creates a new melt pool compared to the shorter 0.5 ms turnaround time. This assumes the laser power and speed are constant across the length of the track. The faster turnaround time is advantageous for decreasing the overall build time but may not be advantageous for a consistent melt pool size. It is possible that cross-sectioning closer to the pad edge will reveal a greater change in melt pool size; however, the position dependence of the melt pool within the pads is clearly diminished by the increased laser turnaround time.

## Conclusions

A set of bare plate nickel alloy 718 single-track (1D) and pad scan (2D) measurements were taken for AM Bench 2022 that includes in situ thermography, EBSD and EDS microstructure, and cross-sectional melt pool size via optical microscopy. The single-track scans included seven cases of laser power, scan speed, and laser spot size. The pad scans used a fixed set of laser parameters and geometry while varying the scan direction between ± X and ± Y directions, matching the scan pattern used for AM Bench 3D builds. Uncertainty budgets for the melt pool size measurements from optical micrographs were provided for the purpose of model validation. There are several key findings from the cross-sectional melt pool size measurements:

- The single-track melt pool depth increased with volumetric energy density while the width did not show a consistent trend within the relatively narrow VED*σ* range studied here. Predictions from eight challenge submissions for width were more accurate than for depth.
- Single tracks and the first track of pad scans showed comparable widths and depths while the average melt pool depth and width for pad scans was higher than single tracks and first tracks of pad scans due to heat buildup. In general, the pad scan depths and widths increased with track number due to heat buildup.
- The pad scan melt pool depth was smaller when the scan direction and gas flow direction were parallel likely due to an increased interaction between the plume by-products and the laser. This was not predicted by the four challenge submissions.
- Cross sections at different locations within pads revealed no trends or weak trends of melt pool size versus position. This was counter to other examples in literature, which show a strong dependence on position near the edges of 2D scans. The laser turnaround time for the pads was much slower (≥ 5.0 ms) than other examples, which allowed for more cooling between the end of one track and the start of the next track.

## Figures and Tables

**Table 1:** List of relevant experiments, measurements, and dataset references for AM Bench 2022

Experiment type	Measurement type	Dataset references	Journal references
Single-track and pad scans	Cross-sectional optical microscopy	[7]	This paper
	Thermography	[8]	[9]
	EBSD and EDS	[10]	[11]
3D build	Thermography	[12]	[13]
	EBSD	[14]	[15]

**Table 2:** Base process conditions for single-track scans

laser power	285 W
Laser speed	960 mm/s
Laser spot size (Gaussian diameter)	67 μm
Laser energy distribution	Rotationally symmetric Gaussian
Scan direction (see Fig. 1)	+ X
Track length	10 mm
Inert gas	Argon
Max. oxygen level	< 1 000 ppm
Gas flow speed (Z = 10 mm) and direction	4.3 m/s in − Y
Chamber pressure	95 kPa ± 5 kPa
Substrate and chamber temperature	23.5 °C ± 1 °C
Laser incidence angle	5° ± 0.5°

The same laser power, speed, and spot size were used for pad scans. The coverage factor for chamber pressure, temperature, and laser incidence angle is *k* = 1. The machine reference frame is as follows. *Z* is the build direction, *X* is the recoating direction, and *Y* is perpendicular to *Z* and *X*. Viewed from the front of the machine, + X is to the right, and + Y is from front to back

**Table 3:** Laser parameters for different cases of single tracks

	Case number	Laser power (W)	Scan speed (mm/s)	Spot size *D4σ* (μm)	VED*σ* = P/v/*σ*^2^ (J/mm^3^)
Baseline	0	285	960	67	1058
Change spot	1.1	285	960	49	1978
	1.2	285	960	82	706
Change speed	2.1	285	1200	67	847
	2.2	285	800	67	1270
Change power	3.1	325	960	67	1207
	3.2	245	960	67	910

Each case was repeated three times. VED*σ* is volumetric energy density based on the laser power, *P*, scan speed, v, and 1*σ* (D4 *σ*/4) laser beam size

**Table 4:** Single-track melt pool measurements: mean, standard deviation, and expanded uncertainty, U (*k* = 2)

Case	Laser power (W)	Scan speed (mm/s)	Spot size (μm)	Width (μm)	SD (μm)	U (k = 2) (μm)	Depth (μm)	SD (μm)	U (k = 2) (μm)	Aspect ratio
0	285	960	67	136.3	2.9	6.2	139.7	1.9	14.1	2.1
1.1	285	960	49	106.2	3.6	5.5	227.2	3.2	22.9	4.3
1.2	285	960	82	141.7	1.8	6.0	102.4	1.1	10.4	1.4
2.1	285	1200	67	112.9	1.7	4.9	109.7	1.7	11.2	1.9
2.2	285	800	67	156.1	4.9	7.7	176.5	2.6	17.9	2.3
3.1	325	960	67	134.3	2.5	6.0	166.1	2.0	16.8	2.5
3.2	245	960	67	129.4	1.6	5.5	116.9	1.2	11.8	1.8

There were six measurements (three tracks, two cross sections per track) per case. The expanded uncertainty follows Ref. [19], and the details are provided in Appendix 3

**Table 5:** Average melt pool measurements for X-pad and Y-pad based on the cross-sectional position and odd versus even track numbers

Pad, cross-sectional position, and odd/even track group	Mean *d*_p_ (μm)	SD *d*_p_ (μm)	Mean *d*_o_ (μm)	SD *d*_o_ (μm)	Mean *w*_p_ (μm)	SD *w*_p_ (μm)	Mean *w*_o_ (μm)	SD *w*_o_ (μm)
X-pad, 0.9 mm, odd	166.0	7.0	82.7	7.3	95.1	6.6	118.2	8.4
X-pad, 0.9 mm, even	162.0	3.9	95.1	11.8	90.0	5.4	95.7	6.8
X-pad, 1.3 mm, odd	163.5	6.1	79.3	7.1	100.0	5.0	127.3	6.6
X-pad, 1.3 mm, even	155.8	4.7	91.8	7.8	86.2	4.2	87.1	4.2
Y-pad, 0.6 mm, odd	175.2	5.9	76.1	7.8	102.3	6.7	131.4	6.2
Y-pad, 0.6 mm, even	120.0	10.2	88.1	6.7	82.3	4.5	81.3	6.3
Y-pad, 1.5 mm, odd	175.1	6.7	82.3	6.7	100.8	9.0	123.9	8.1
Y-pad, 1.5 mm, even	123.5	9.8	88.0	6.3	84.6	7.2	87.2	6.3
Y-pad, 1.9 mm, odd	174.0	7.1	83.3	9.3	98.0	7.8	122.2	7.8
Y-pad, 1.9 mm, even	124.7	10.7	89.2	8.9	83.0	3.5	89.0	4.2
Y-pad, 2.9 mm, odd	170.1	7.2	87.6	6.8	91.8	9.7	109.7	7.3
Y-pad, 2.9 mm, even	137.1	7.7	91.1	8.8	86.6	5.1	101.7	6.9

The uncertainty budget and expanded uncertainty (U) are included in Appendix 4

**Fig. 12:**
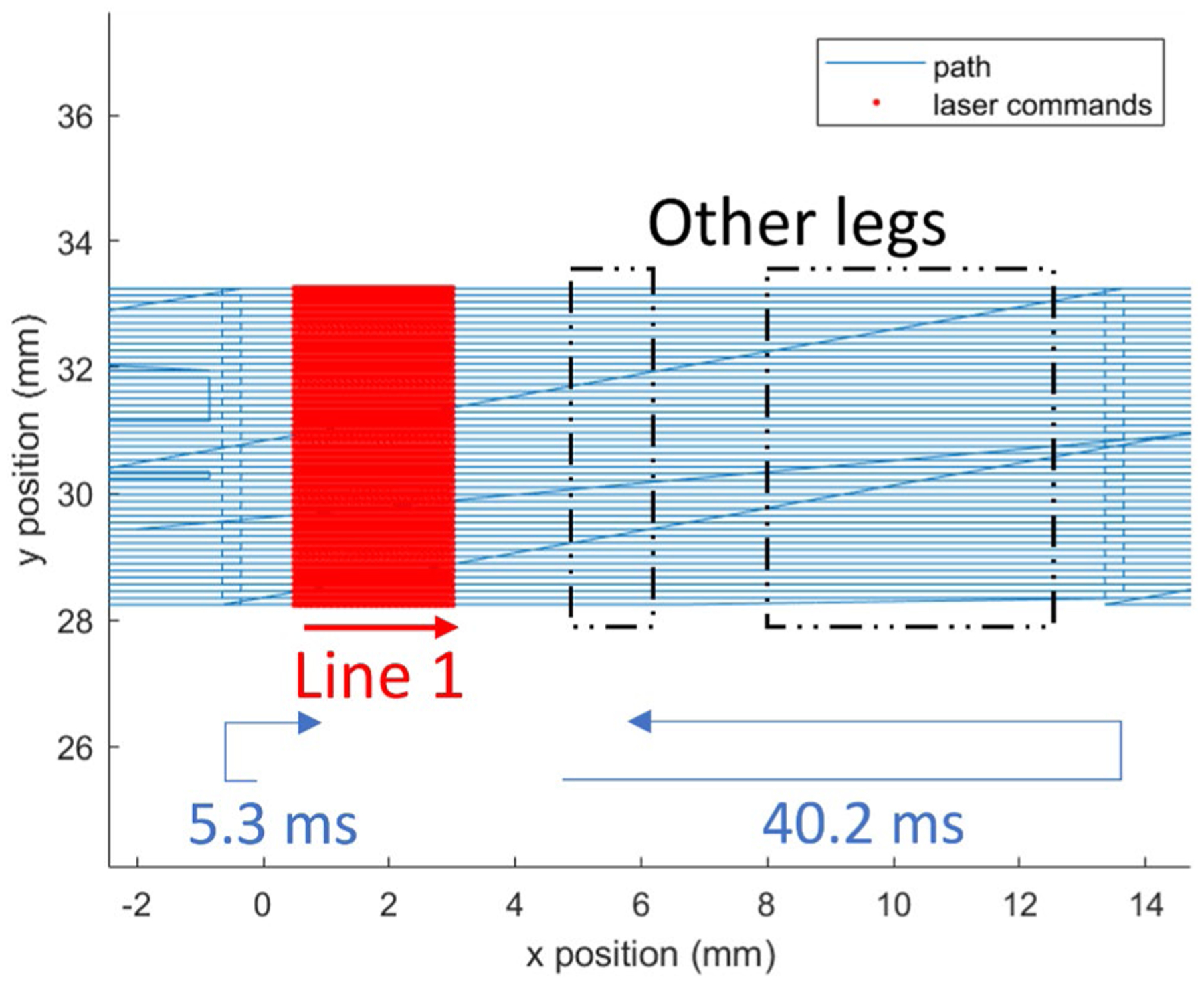
Laser path (light blue) and when the laser power is on (red) showing the X-pad and the other two legs that are traversed causing the longer turnaround time on the right side of the pad geometry. The pad geometry and timing replicate the medium sized leg in the 3D build

## Data Availability

Optical micrographs and melt pool measurements are published online: Weaver, Jordan, Deisenroth, David, Mekhontsev, Sergey, Lane, Brandon, Levine, Lyle, Yeung, Ho (2022), AM Bench 2022 Measurement Results Data: Optical Microscopy of Laser-scanned Single Tracks and Pads (AMB2022–03), National Institute of Standards and Technology, https://doi.org/https://doi.org/10.18434/mds2-2718.

## Footnotes

1 Certain equipment, instruments, software, or materials are identified in this paper in order to specify the experimental procedure adequately. Such identification is not intended to imply recommendation or endorsement of any product or service by NIST, nor is it intended to imply that the materials or equipment identified are necessarily the best available for the purpose.

## Appendix 1

See Table 6.

**Table 6:** Vendor supplied chemical composition for nickel alloy 718 plate

Fe	Ni	Cr	Nb	Mo	Ti	Al	Co
Balance	51.54	18.27	5.15	2.90	1.04	0.57	0.79
Cu	Si	Mn	Ta	C	S	P	B
0.04	0.08	0.14	0.01	0.05	0.0002	0.01	.002

No uncertainties were provided by the vendor. Values in this table are taken from vendor-supplied data sheets, which utilized combustion/infrared for C and S, wavelength-dispersive X-ray fluorescence (WDXRF) for Mn, P, Si, Cr, Ni, Mo, Cu, Nb, Co, Al, Ti, and optical emission spectroscopy (OES) for B. All composition measurements are in mass (weight) percent

## Appendix 2

See Fig. 12

## Appendix 3

The uncertainty budget for the single-track depth and width measurements follows the work by Lane et al. [19]. This includes four components of uncertainty: optical resolution, user boundary selection, variability along the track length, and the standard uncertainty of the mean from repeat measurements. The optical resolution is estimated at 0.5 μm. The user variability in selecting the boundaries from Ref. [19] (± 6 pixels) can be applied since the pixel size and measurement procedures are nearly identical. The variability of the depth and width along the track length are estimated to be 5% and 2% of the mean, respectively. This ignores any transient behavior at the very start and end of the tracks (e.g., < 1 mm). These estimates are limited to bare plate scans with stable melting (i.e., no powder, no unstable keyhole melting). See Ref. [19] for additional details regarding variability along the track length. The standard uncertainty of the mean is determined as $t_{1-a/2\frac{\sigma }{\sqrt{n}},}$ where *t* is a sling factor from the Student t-distribution for a probability of *α* = 68%, *σ* is the standard deviation, and *n* is the number of measurements. The combined uncertainty is root sum of squares, and the expanded uncertainty (U) is twice the combined uncertainty using a coverage factor *k* = 2 [26]. The results are all listed in Table 7.

**Table 7:** Uncertainty budget for single-track melt pool depth and width measurements

	Unit	Case 0	Case 1.1	Case 1.2	Case 2.1	Case 2.2	Case 3.1	Case 3.2	Prob. Distr	Analysis type
*Components of standard uncertainty for depth and width*										
Optical resolution	(μm)	0.50	0.50	0.50	0.50	0.50	0.50	0.50	Norm	Type B
User selection	(μm)	0.41	0.41	0.41	0.41	0.41	0.41	0.41	Norm	Type B
*Components of standard uncertainty for depth*										
Variability along track, depth	(μm)	6.99	11.36	5.12	5.49	8.82	8.30	5.85	Norm	Type B
Standard uncertainty of the mean	(μm)	0.87	1.45	0.52	0.78	1.18	0.92	0.55	Norm	Type A
*Components of standard uncertainty for width*										
Variability along track, depth	(μm)	2.73	2.12	2.83	2.26	3.12	2.69	2.59	Norm	Type B
Standard uncertainty of the mean	(μm)	3.09	2.76	3.02	2.47	3.87	2.99	2.76	Norm	Type A
*Combined uncertainties for depth*										
Combined standard uncertainty	(μm)	7.07	11.47	5.19	5.58	8.92	8.38	5.91		
Expanded uncertainty, U (*k* = 2)	(μm)	14.14	22.94	10.38	11.16	17.85	16.76	11.82		
*Combined uncertainties for width*										
Combined standard uncertainty	(μm)	3.09	2.76	3.02	2.47	3.87	2.99	2.76		
Expanded uncertainty, U (*k* = 2)	(μm)	6.17	5.52	6.03	4.94	7.74	5.98	5.52		

## Appendix 4

The uncertainty budget for pad scan measurements contains the same four components of uncertainty as the single-track measurements with slight modifications. The optical resolution is the same: 0.5 μm. The user boundary selection was increased from ± 6 pixels to ± 10 pixels (0.69 μm). The remelting of tracks may increase the user selection uncertainty because the contrast between melt pools is less than the contrast between a single melt pool and the bare plate microstructure. For comparison, Ref. [3] reports an average user selection error of 2.6 μm for similarly sized melt pools. However, the melt pools were generated with powder present on top of additively manufactured substrates, which is an even more challenging scenario for user selection. In that scenario, the substrate surface contains waviness, and melt pools are present in the substrate material reducing the contrast compared to the present case. Hence it is reasonable that the user selection for bare plate pads is less than the value reported in Ref. [3] for single layers with powder on AM substrates. There is greater variation in pad scan melt pool size compared to single tracks due to the variation in thermal history; however, the average measurements are defined at each cross-sectional position rather than for the entire pad. The cross-sectional position standard uncertainty (*k* = 1) is estimated to be ± 0.2 mm based on user experience. The melt pool dimensions did not change drastically with cross-sectional position, which is believed to be partly due to the longer laser turnaround time (see Discussion). Therefore, a variability of 5% of the mean of each measurement, similar to single-track scans, is reasonable. Serial sectioning is required to determine a better uncertainty for the variability at each cross-sectional position. The average and standard deviation was determined at each cross-sectional position for odd and even track numbers. This means the standard uncertainty of the mean is determined from the multiple tracks within a pad on each cross section and not multiple repeated cross sections. Multiple pad cross sections at the same location were not attempted due to limited resources. The uncertainties were combined and expanded in the same way as described for single-track measurements. The values are listed in Tables 8 and 9.

**Table 8:** Pad depth and overlap depth uncertainty budget

	Depth variability	Depth, Std. uncertainty of mean	Depth, Exp. uncertainty, U (*k* = 2)	Overlap depth variability	Overlap depth, Std. Unc. of mean	Overlap depth Exp. uncertainty U (*k* = 2)
Type	Type B	Type A		Type B	Type A	
Prob. Distr	Norm	Norm		Norm	Norm	
Units	μm	μm	μm	μm	μm	μm
X-pad, 1.3 mm, odd	8.30	1.45	16.94	4.13	1.58	9.02
X-pad, 1.3 mm, even	8.10	0.85	16.38	4.75	2.56	10.93
X-pad, 0.9 mm, odd	8.17	1.26	16.63	3.97	1.54	8.68
X-pad, 0.9 mm, even	7.79	1.01	15.80	4.59	1.68	9.93
Y-pad, 1.9 Mm, odd	8.70	2.12	17.99	4.17	2.92	10.32
Y-pad, 1.9 mm, even	6.23	3.35	14.26	4.46	2.78	10.65
Y-pad, 0.6 mm, odd	8.76	1.77	17.96	3.80	2.43	9.19
Y-pad, 0.6 mm, even	6.00	3.19	13.70	4.41	2.10	9.91
Y-pad, 2.9 mm, odd	8.51	2.17	17.64	4.38	2.13	9.89
Y-pad, 2.9 mm, even	6.86	2.43	14.65	4.55	2.76	10.78
Y-pad, 1.5 mm, odd	8.75	1.99	18.04	4.12	2.10	9.40
Y-pad, 1.5 mm, even	6.17	3.09	13.91	4.40	1.97	9.79

The resolution uncertainty is 0.5 μm. The user selection uncertainty is 0.69 μm. Both are type B uncertainties. These were used in the combined uncertainty before using a coverage factor of *k* = 2 to determine the expanded uncertainty (U). These three things were not included in the table to save space

**Table 9:** Pad width and overlap width uncertainty budget

	Width variability	Width, Std. uncertainty of mean	Width, Exp. uncertainty, U (*k* = 2)	Overlap width variability	Overlap width, Std. Unc. of mean	Overlap width Exp. uncertainty U (*k* = 2)
Type	Type B	Type A		Type B	Type A	
Prob. Distr	Norm	Norm		Norm	Norm	
Units	μm	μm	μm	μm	μm	μm
X-pad, 1.3 mm, odd	4.75	1.38	10.05	5.91	1.81	12.48
X-pad, 1.3 mm, even	4.50	1.16	9.45	4.79	1.46	10.16
X-pad, 0.9 mm, Odd	5.00	1.03	10.35	6.36	1.43	13.16
X-pad, 0.9 mm, even	4.31	0.91	8.97	4.36	0.92	9.07
Y-pad, 1.9 mm, odd	4.90	2.32	10.98	6.11	2.44	13.26
Y-pad, 1.9 mm, even	4.15	1.11	8.76	4.45	1.33	9.44
Y-pad, 0.6 mm, odd	5.12	2.02	11.13	6.57	1.95	13.81
Y-pad, 0.6 mm, even	4.11	1.42	8.87	4.06	1.97	9.19
Y-pad, 2.9 mm, odd	4.59	2.89	10.98	5.48	2.28	12.00
Y-pad, 2.9 mm, even	4.33	1.59	9.38	5.08	2.15	11.17
Y-pad, 1.5 Mm, odd	5.04	2.69	11.56	6.19	2.54	13.50
Y-pad, 1.5 mm, even	4.23	2.27	9.74	4.36	1.98	9.73

The resolution uncertainty is 0.5 μm. The user selection uncertainty is 0.69 μm. Both are type B uncertainties. These were used in the combined uncertainty before using a coverage factor of *k* = 2 to determine the expanded uncertainty (U). These three things were not included in the table to save space
